# Supplementary figures and images for: Increased atherosclerosis and expression of inflammarafts in macrophage foam cells in AIBP-deficient mice
Source: Sci Rep. 2026 Feb 7;16:7645. doi: 10.1038/s41598-026-39113-2 (PMC12936166; doi:10.1038/s41598-026-39113-2)

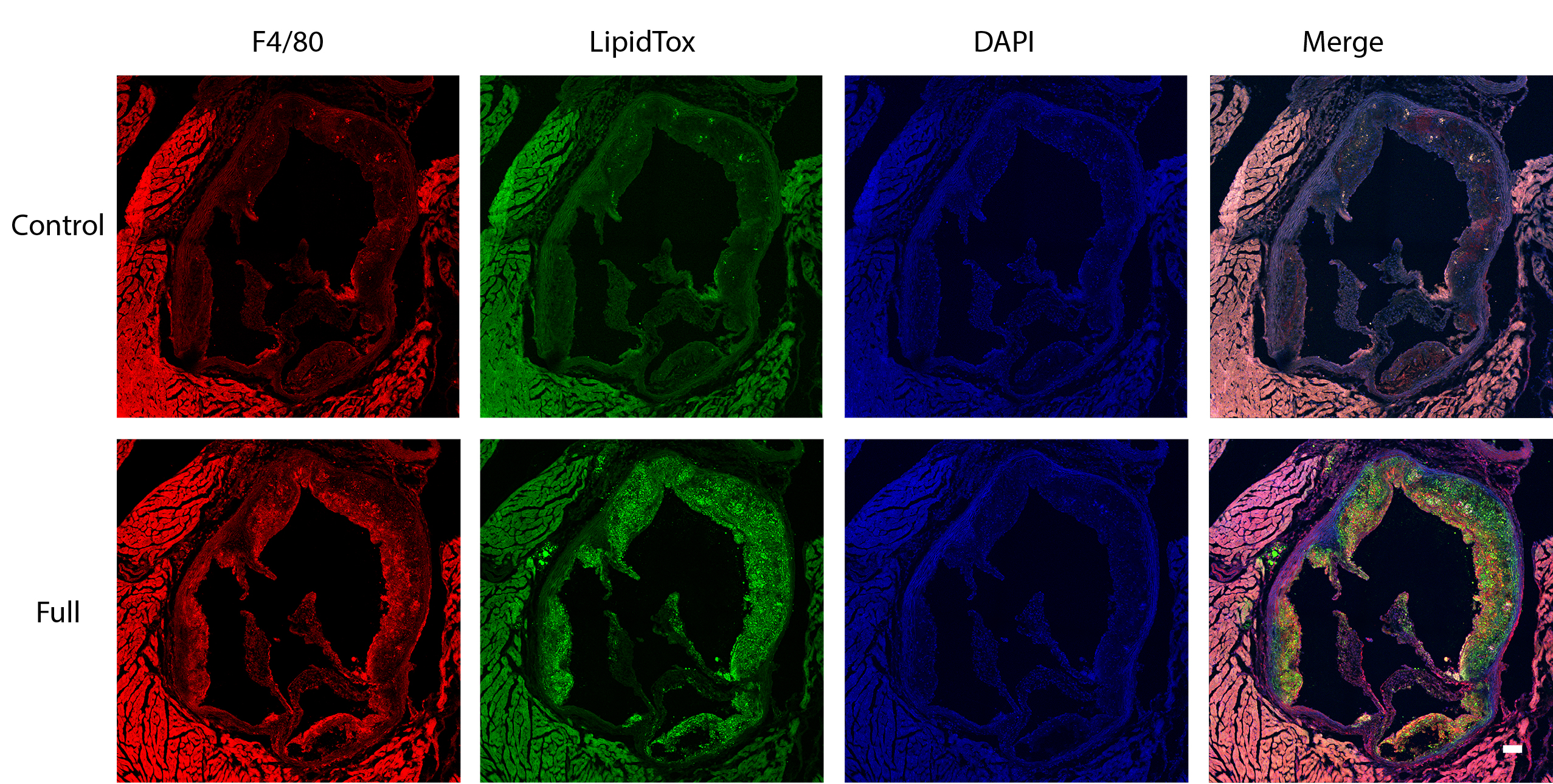

Supplement: Supplementary file 1 — Supplementary Material 1 [file 41598_2026_39113_MOESM1_ESM.png]

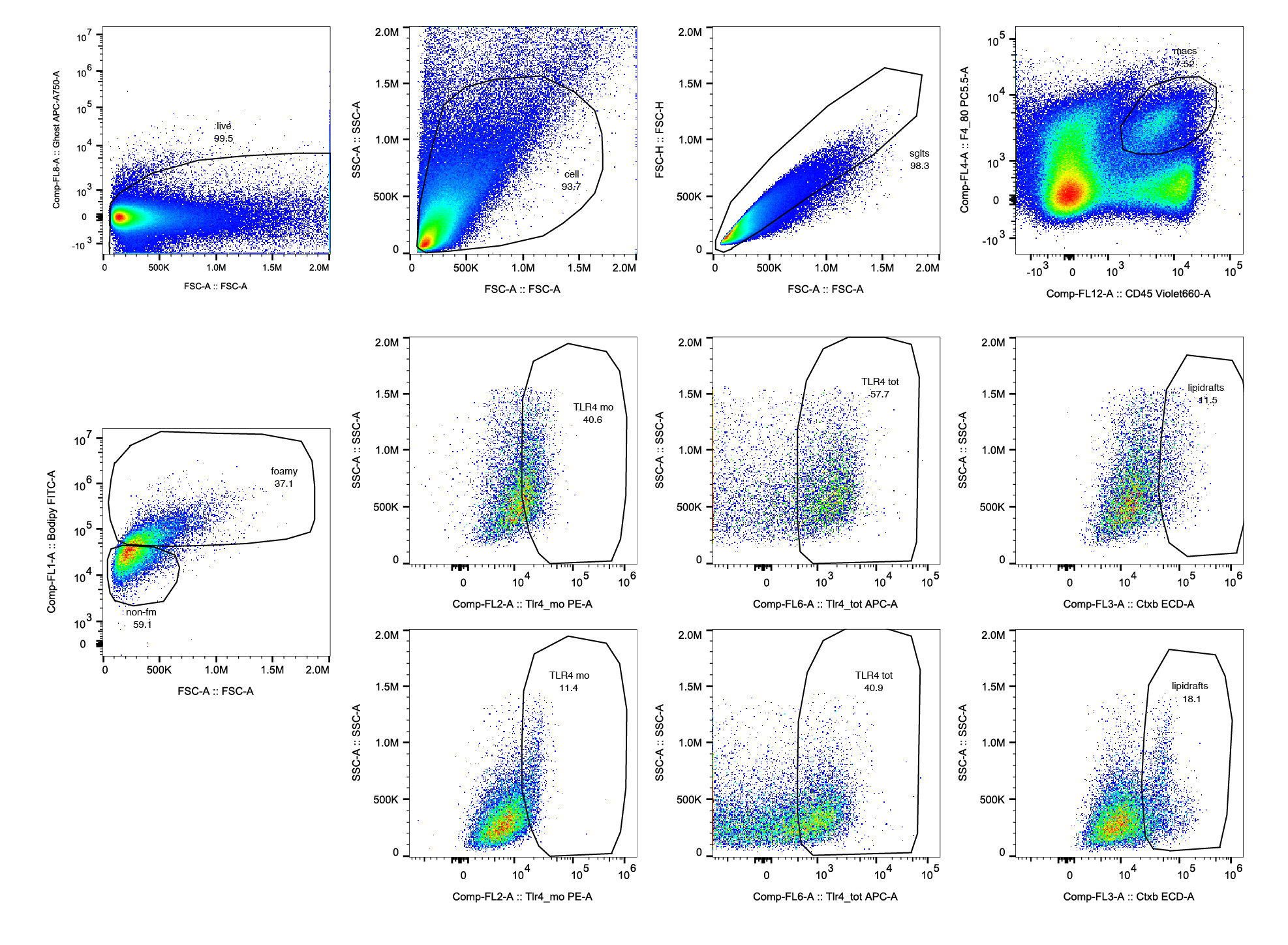

Supplement: Supplementary file 2 — Supplementary Material 2 [file 41598_2026_39113_MOESM2_ESM.png]

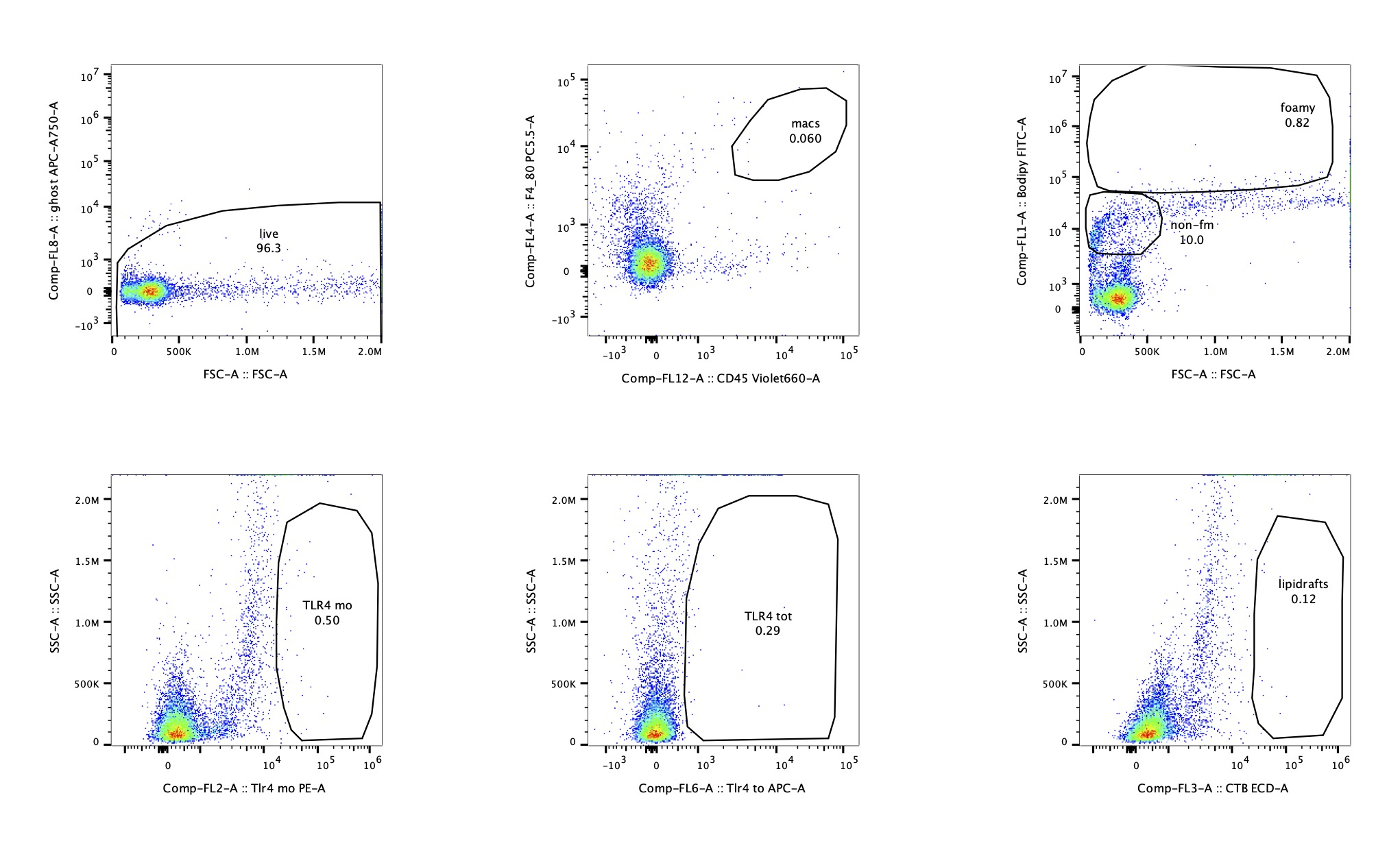

Supplement: Supplementary file 3 — Supplementary Material 3 [file 41598_2026_39113_MOESM3_ESM.png]

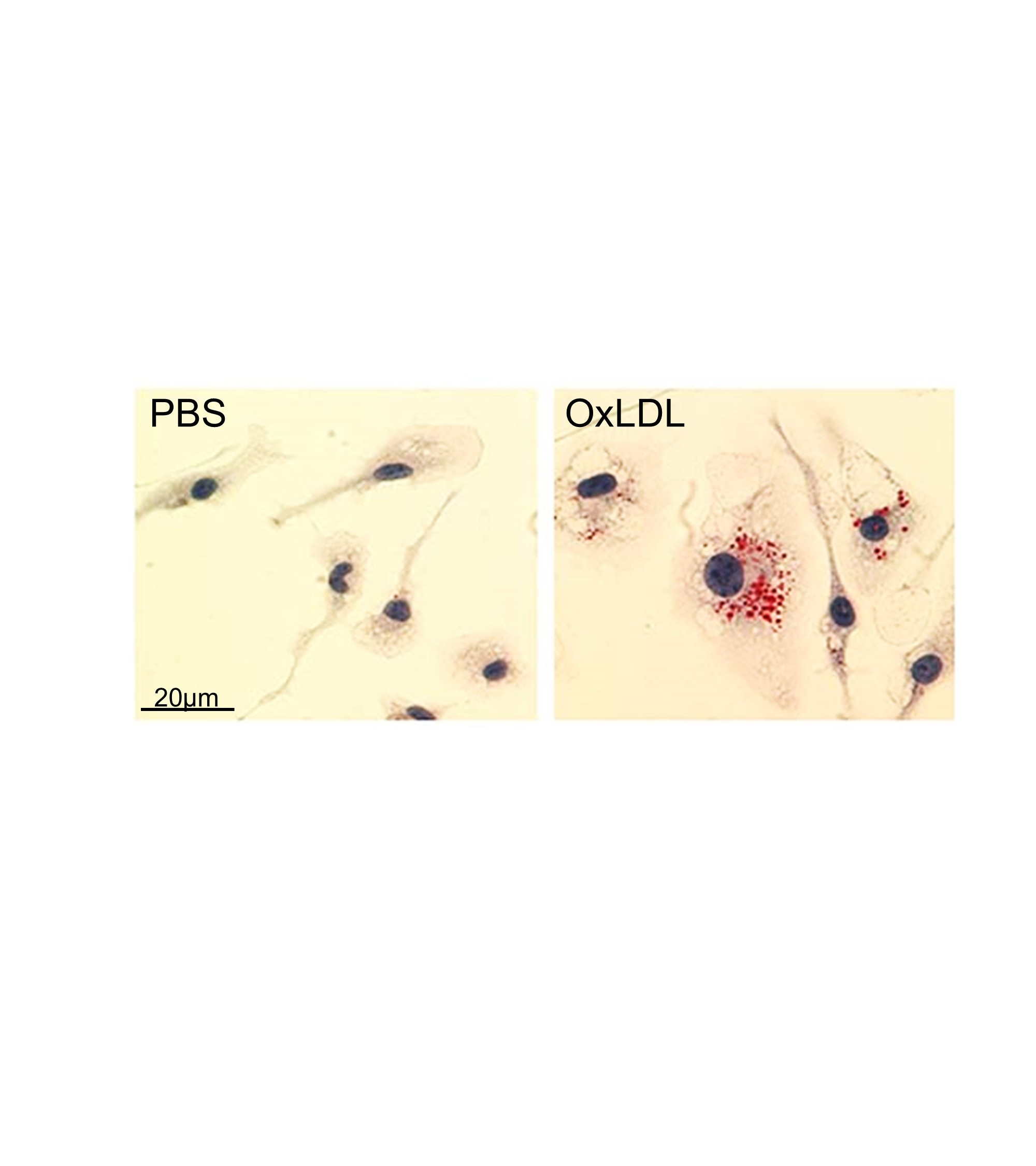

Supplement: Supplementary file 4 — Supplementary Material 4 [file 41598_2026_39113_MOESM4_ESM.png]
