# Supplementary material for: Increased atherosclerosis and expression of inflammarafts in macrophage foam cells in AIBP-deficient mice
Source: Sci Rep. 2026 Feb 7;16:7645. doi: 10.1038/s41598-026-39113-2 (PMC12936166; doi:10.1038/s41598-026-39113-2)
Supplement: Supplementary file 7 — Supplementary Material 7 [file 41598_2026_39113_MOESM7_ESM.pdf]

Li *et al*. Increased atherosclerosis and expression of inflammarafts in macrophage foam cells in AIBP-deficient mice

Supplemental Figure and Table Legends

Supplemental Figure S1. Negative control of immunostaining. A. Negative control panel stained by fluorophore-conjugated secondary antibody and DAPI only. B. Full staining panel stained with anti-F4/80 antibody, secondary antibody, LipidTOX, and DAPI. Images were acquired in all 3 color channels. The control shows non-specific autofluorescence from the cardiac muscle tissue.

Supplemental Figure S2. Gating strategy for flow cytometry analysis of expression of TLR4 dimers and lipid rafts. Aortic single-cell suspensions from *Apoa1bp*$^{-/-}$*Ldlr*$^{-/-}$ and *Ldlr*$^{-/-}$ mice fed a 16-week high-fat diet were gated for BODIPY-high foamy and BODIPY-low non-foamy, CD45^{+} F4/80^{+} macrophages. The percentage of TLR4 dimers was calculated from geometric mean fluorescence intensities of PE-conjugated TLR4/MD2 antibody (monomers) and APC-conjugated TLR4 antibody (total). Lipid rafts were calculated from the geometric mean fluorescence intensity of AF594-conjugated cholera toxin B subunit.

Supplemental Figure S3. Unstained controls used in gate selection of flow cytometry. Aortic single-cell suspensions from *Apoa1bp*$^{-/-}$*Ldlr*$^{-/-}$ and *Ldlr*$^{-/-}$ mice fed a 16-week high-fat diet were used for unstained cells and APC-Cy7^{+} dead cells. After compensation, unstained cells were used as negative controls for gating.

Supplemental Figure S4. Validation of lipid droplet formation in BMDMs. After 24-hour incubation with 20 μg/mL OxLDL, BMDMs were stained with Oil Red O and counterstained with hematoxylin.

Supplemental Table T1. Metadata of RNA-seq data.

Supplemental Table T2. Primers for real-time qPCR.
